# Core functions and forms of Bright IDEAS: A multi-methods evaluation of the adoption of an evidence-based psychosocial training program through iterative adaptation

**DOI:** 10.3389/frhs.2022.928580

**Published:** 2022-11-04

**Authors:** Demetria M. McNeal, Olle Jane Z. Sahler, Robert B. Noll, Diane L. Fairclough, Megan E. Voll, Shubha Bhat, Elaine H. Morrato

**Affiliations:** ^1^Division of General Internal Medicine, University of Colorado School of Medicine, Aurora, CO, United States; ^2^Adult and Child Consortium for Health Outcomes Research and Delivery Science, University of Colorado Anschutz Medical Campus, Aurora, CO, United States; ^3^Division of Pediatric Hematology/Oncology, University of Rochester School of Medicine and Dentistry, Golisano Children's Hospital, Rochester, NY, United States; ^4^Department of Pediatrics, School of Medicine, University of Pittsburgh, Pittsburgh, PA, United States; ^5^Colorado School of Public Health, University of Colorado Anschutz Medical Campus, Aurora, CO, United States; ^6^Skaggs School of Pharmacy and Pharmaceutical Sciences, University of Colorado Anschutz Medical Campus, Aurora, CO, United States; ^7^Parkinson School of Health Sciences and Public Health, Loyola University Chicago, Maywood, IL, United States

**Keywords:** dissemination, cancer survivorship, psychosocial intervention, core functions and form, intervention-implementation interface

## Abstract

**Background:**

Despite efforts to widely disseminate interventions designed to increase access to quality supportive care to pediatric cancer patients and their families, many of these interventions fail to meet expectations once deployed in real-life clinical settings. This study identifies the functions and forms of Bright IDEAS: Problem-Solving Skills Training, an evidence based psychosocial intervention for caregivers of children recently diagnosed with cancer, to identify pragmatic program adaptations in its real-world clinical implementation. We compare intervention adoption before and after adaptations to the Bright IDEAS training program as part of a national training program designed to disseminate the intervention.

**Methods:**

209 pediatric psychosocial oncology practitioners representing 134 unique institutions were trained during 10 in-person 8-hour workshops (2015–2019). Functions and forms of Bright IDEAS were identified, and adaptations made to the training agenda and curriculum based on practitioner feedback following implementation in local institutions. Mixed method evaluation included longitudinal surveys at 6- and 12-months post training; and qualitative interviews among a subgroup of practitioners (*N* = 47) to understand and compare perspectives on intervention adoption and barriers to implementation before and after adaptations to the Bright IDEAS training program. The RE-AIM framework was used to guide dissemination evaluation.

**Results:**

A total of four adaptations were tailored to the identified forms of the intervention: case studies; pre-training reading materials; training videos; and letters of institutional support from primary supervisor. Pre- and post-training adaptations to the Bright IDEAS training program were mapped to RE-AIM constructs. Quantitative findings demonstrate that adaptations appeared to improve adoption and usage overall.

**Conclusion:**

This study provides insight into how contextual factors influence psychosocial practitioners' capacity to adopt, implement, and maintain Bright IDEAS in the clinical setting. This study demonstrates the use of real-time stakeholder feedback to guide intervention translation from research to practice settings.

## Background

### Bright IDEAS

Problem-Solving Skills Training (Bright IDEAS) is an evidence-based cognitive behavioral therapy that has over 25 years of empirical evidence demonstrating a decrease in negative affectivity (mood, depression, post-traumatic stress symptoms) in mothers of children with recently diagnosed cancer (1–4). Problem-solving therapy (PST) is a cognitive-behavioral approach developed to treat depression and anxiety in adults (5). The decision to call the intervention Problem-Solving Skills Training rather than therapy was aimed at making the intervention acceptable to distressed parents, who did not feel that they required “therapy.”

Multisite randomized controlled trials (RCTs) funded by the NIH/NCI over 25 years showed that learning the 5-step Bright IDEAS paradigm improves problem-solving skills and improved problem-solving skills led to decreased depression, improved mood, and fewer symptoms of posttraumatic stress (1, 4). Specifically, when compared to a nonspecific behavioral intervention, which provided the same time and attention from research assistants and focused on non-judgmental support and expression of feelings, participants of Bright IDEAS, at the 3-month follow up (T3) showed significant improvements in mood (−2.78 vs. −9.33, *p* ≤ 0.009), anxiety (−0.14 vs. −0.54, *p* ≤ 0.001), and post-traumatic stress (−2.27 vs. −4.01, *p* = 0.12) (6). Additionally, Bright IDEAS, when compared to control, had the greatest impact on improving constructive problem solving, accounting for 40% of the difference in mood scores between the two groups (1). In a two-arm randomized clinical trial of usual psychosocial care (UPC) as the control condition vs. UPC + Bright IDEAS as the intervention condition, mothers that received UPC + Bright IDEAS reported significantly enhanced problem-solving skills and significantly decreased negative affectivity (2).

**Figure 1:**
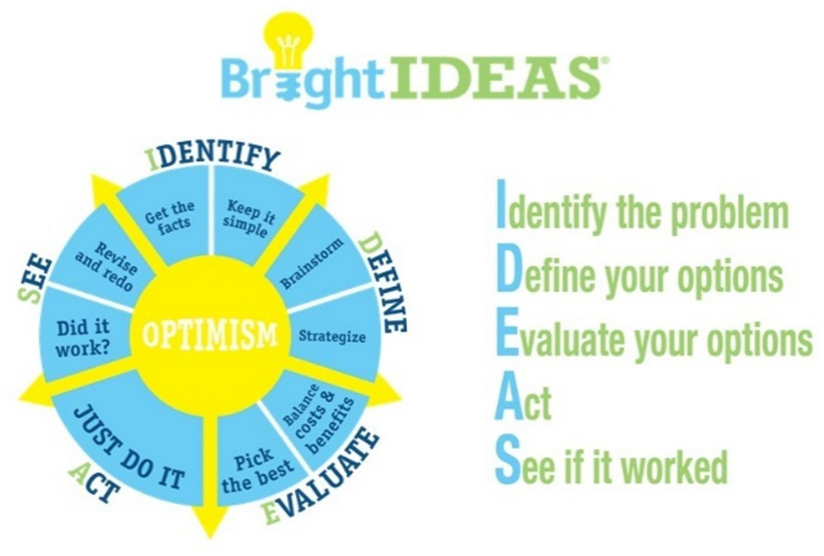
Bright IDEAS pneumonic.

The Bright IDEAS intervention is designed to empower individuals to manage adverse situations by using constructive coping strategies. It is a five-step cognitive-behavioral intervention based on the theoretical underpinnings of established problem-solving therapy (PST) (5, 7). Bright IDEAS represents a mnemonic (Figure 1). Bright signifies the concept of optimism (i.e., positive problem orientation), which is essential to successful problem-solving. The letters in the word “IDEAS” each stand for one of the five steps in the problem-solving process: I (Identify the problem), D (Define possible options), E (Evaluate your options—pros and cons of each option), A (Act—create an action plan based on D and E and do it), and S (See if it worked). If the plan did not work, review the options and devise a Plan B.

No constraints are placed on the type of problem or challenge that Bright IDEAS can address. Of note, the majority of problems selected by the caregivers who participated in Bright IDEAS efficacy studies were not related to pediatric cancer (1–3). Optimum engagement is gained by focusing on problems the caregiver identifies as particularly relevant to him or her and walking through each of the steps. Clinical trials have shown that Bright IDEAS is acceptable to caregivers when taught in 6–8 and 30–60-min face-to-face sessions (1) and more effective over time than one of the most common forms of psychosocial support, non-directive supportive counseling (6).

The National Cancer Institute (NCI) designated Bright IDEAS an Evidence-Based Cancer Control Program (EBCCP; formerly, Research-Tested Intervention Program), in 2010. The EBCCP is a public-facing searchable database of evidence-based cancer control programs designed to provide program planners and health professionals easy and immediate access to research-tested materials. NCI tracking statistics indicate the Bright IDEAS webpage received 370 views on average over an 8-year period (averaging 2.7 min per page); and 40 CDs of the intervention materials were requested to be mailed. These findings suggest that, despite NCI endorsement, public availability, and considerable evidence supporting its efficacy, the leap from research protocol to standard clinical care was minimal.

There is increasing urgency to address the gap between the generation of new knowledge and empirical evidence, and its application to routine clinical care (8, 9). This urgency is fueled, in part, by the many interventions that fail to meet expectations once deployed in real-life clinical settings. Typically, interventions are tested under controlled conditions that are unlike the clinical practice settings in which they are deployed. As a result, many interventions lack full consideration of the local and contextual factors that ultimately affect intervention implementation (9, 10).

To overcome intervention failure in clinical settings, the field of dissemination and implementation (D&I) science has called attention to the importance of adapting existing evidence-based interventions to improve their fit in new contexts (11, 12). A critical first step in adaptation is to identify core functions (purposes) and forms (activities). Core functions represent the central purpose of the change processes that the health intervention seeks to facilitate. The forms of an intervention are the specific strategies or activities that can be customized to local contexts to carry out the core functions. Core functions should be considered unchallengeable as they are the essential mechanisms responsible for intervention efficacy. Adaptation at the form level, however, allows flexibility for organizations to tailor an intervention to their specific setting and situtaion (11, 12). Ideally, an intervention's core functions and forms align with health system and patient needs at the clinical level to ensure both the integrity of the intervention and its successful implementation.

Despite efforts to scale up cancer control interventions, there are limited data assessing the adoption of NCI-recognized survivorship and supportive care EBCCPs into clinical practice (13). Specifically, adaptation of Bright IDEAS had not been considered previously. This study examines the core functions and forms of Bright IDEAS and the impact of adaptations to the training program on its real-world clinical use based on feedback from participants in this natural experiment of an NCI-supported dissemination training grant. The goal of the grant was to increase national awareness of Bright IDEAS, train providers on how to deliver Bright IDEAS, and facilitate adoption amongst the approximately 200 pediatric oncology centers operating in North America. The multi-methods evaluation presented in this paper, involving both survey and qualitative assessment, seeks to elucidate barriers to adoption, implementation, and maintenance of a psychosocial intervention in diverse real-world pediatric oncology practice settings.

## Methods

### Training format

An NCI training grant (R25 CA65520) was awarded to train 200 pediatric psychosocial oncology professionals throughout the United States by conducting 10 interactive in-person training workshops between October 2015 and September 2019. Practitioner recruitment for the workshops was conducted in partnership with national professional organizations intimately involved in pediatric oncology: Children's Oncology Group (COG), Association of Pediatric Oncology Social Workers (APOSW), the Association of Pediatric Hematology/Oncology Nurses (APHON), and the Society of Pediatric Psychology (SPP).

The 1½-day training workshops were held in conjunction with association national meetings and endorsed through co-advertising. The workshops included summary information about the three large multi-site randomized controlled trials conducted to date demonstrating the efficacy of Bright IDEAS; role plays to observe and practice administration of the intervention; and in-depth discussions about implementation at an attendee's specific home institution. The original training agenda was modeled after the research training protocol used in clinical trials, which included a clinician's manual that detiled the basic approach and discrete steps of Bright IDEAS, a brief user's manual summarizing three steps, and worksheets (14). In the pre-adaptation phase we delivered the training curriculum based on the research protocol used in clinical trials. Post-adaptation phase we delivered the training curriculum that was informed by real-world clinical practice feedback from participants. Adaptations to the training program were made to mitigate perceived barriers to clinical application of Bright IDEAS in institutional settings. Workshop participants received up to $1,000 to reimburse expenses associated with travel and lodging.

### Study population

All training participants (*N* = 209; pediatric oncology psychosocial professionals representing 134 unique institutions) were electronically surveyed at 6- and 12-months post workshop training. Survey response rate was 85.6% (*n* = 179) at 6 months and 72.2% (*n* = 149) at 12 months.

In addition, a subset of trainees were purposively sampled (*N* = 47, 24.4% of participants) and interviewed between January 2017 and March 2020. Interviews represented a range of post-training workshop experience before and after adaptations were made to the training workshop agenda: *Pre-adaptation*: Wave 1- more than 12 months since training (*N* = 11), Wave 2- between 6 and 12 months (*N* = 9), and Wave 3- less than 6 months (*N* = 10). *Post-adaptation*: Wave 1- more than 12 months since training (*N* = 6), Wave 2- between 6 and 12 months (*N* = 4), and Wave 3- less than 6 months (*N* = 4). The pre-adaptation group participated in the workshops that delivered the original training curriculum as used in the clinical research studies. The post-adaptation group participated in the workshops that delivered the adapted training curriculum designed to be more relevant for real-world clinical practice. We define the core intervention as the IDEAS psychosocial behavioral intervention pneumonic. The intervention materials were streamlined in their delivery, not in the content they conveyed.

Practitioners were contacted *via* email and invited to participate in a 30-minute telephone interview. In total, 106 professionals were contacted, 68 responded to the study invitation (64% response), and 44 were scheduled (65% participation) for interviewing allowing up to three contact attempts. A $25 gift card was offered for participation.

The project was approved by the Colorado Combined Institutional Review Board (COMIRB).

### Evaluation methods

The RE-AIM framework, which is recognized by the NCI as a leading implementation framework in cancer control research, was used to guide the evaluation process (15). We intentionally focused on three of the five dimensions of RE-AIM given the clinical and translational stage of the Bright IDEAS program: adoption, implementation, and maintenance. We did not focus on reach in this study as the goal of the training grant was directed at providers (target adopters) of the intervention and dissemination reach was not focused on the beneficiaries of the intervention. Effectiveness was not assessed because it had been previously established as Bright IDEAS has been an NCI EBCCP for greater than 10 years (1–3, 6). Therefore, facilitating and understanding adoption, implementation, and maintenance of Bright IDEAS were the primary objectives of the training grant.

Quantitative survey outcome measures and qualitative codes were aligned with constructs of the three RE-AIM dimensions: adoption, implementation, and maintenance. Outcome measures included: intervention satisfaction (e.g., *likelihood I will recommend Bright IDEAS to a colleague*); barriers to adoption (e.g., *lack time in clinic*); implementation (e.g., *lack of opportunity (clients)*; and maintenance of Bright IDEAS in clinical practice (e.g., *reimbursement and/or insurance issues)*.

Supplementary materials provide the survey instrument and semi-structured interview guide. The guide was pilot tested with a small sample of psychosocial providers (*N* = 5) and changes were made based on feedback. Semi-structured interviews (range: 24–47 min per interview) were conducted over the telephone by the first author (DMM) who had no prior relationship with any of the respondents. All interviews were audio-recorded and transcribed verbatim.

### Data analyses

Survey responses were coded into REDCap (16) secure web application for building and managing online surveys and databases and analyzed using SAS. Descriptive analyses were performed to summarize demographic characteristics of training participants and outcome measures of Bright IDEAS use, satisfaction, and implementation barriers at 6- and 12-months post training. Outcome measures were stratified into pre-adaptation and post-adaptation time periods and compared using Chi-square and two sample *t*-tests statistical test.

Analyses of the in-depth interviews were completed using data analysis package ATLAS.ti 8.0 (Scientific Software Development GmbH, Berlin, Germany) for coding by study authors (DMM, SB) who are PhD and PharmD trained researchers with experience in qualitative methods, health services research, and D&I science. All the transcripts were double coded. The coders familiarized themselves with the data by carefully reading the transcripts. They then deductively coded the data using the constructs of the three RE-AIM dimensions: adoption, implementation, and maintenance (17, 18). Discrepancies were resolved through discussion. Interviewer and analytic biases were managed during regular analysis meetings among all authors. Two study authors (DMM, SB) engaged in regular discussion of cases throughout the data analysis phase to ensure rigor. Transcribed interviews were coded by marked text with phrases indicating content of the discussions (19).

The Consolidated Criteria for Reporting Qualitative Research framework was used to guide the reporting of findings (20). Additionally, criteria for credibility, transferability, and confirmability were used to ensure rigor of this study (21, 22). Strategies used to address credibility included recording interviews and transcribing them; authors frequently discussing findings; encouraging participants to pursue their own line of thinking; and searching the data for conflicting patterns (21, 23). Confirmability was addressed by rigorous review of interview transcripts, the codes used to identify them, and drafts and revisions of the findings (23).

## Results

### Quantitative findings

The core functions and forms of the Bright IDEAS training workshop were identified as part of continuous program evaluation to determine adaptable components for local and clinical context needs (Table 1). The study team took a learning health system approach and conducted an in-person midpoint review in study year three. Based on pre-adaptation evaluation survey findings (*N* = 159) and in-depth interviews (*N* = 33) from workshop participants, implementation barriers or facilitators were identified, and new implementation strategies developed to mitigate barriers identified by study participants. As a result, four adaptations (Table 2) were made to the forms of the training program to facilitate transition from a research context to a clinical care context. Adaptations are described in Table 2 and involved the added requirement of institutional support to attend the training; changes in pre-workshop study materials; case study role playing in the training workshop; and added guidance to improve clinical workflow integration.

**Table 1:** Core functions and forms of Bright IDEAS.

**Motivating need**	**Intervention design and implementation**	
**Problem to be addressed**	**Core functions (standardized)**	**Forms (tailored)**
1. Identify caregivers in distress who might benefit most from Bright IDEAS Mothers/caregivers of children with cancer experience significant distress associated with their children's diagnosis and treatment.	A.Target ideal candidates for intervention B.Offer psychosocial support to mothers/caregivers of children newly diagnosed with cancer	Case studies and role-play
2. Desire for evidence-based interventions to improve quality of clinical care Psychosocial care for mothers/caregivers is not consistently driven by scientific evidence or supported by local institutions	A.Provide synchronous skill-building training guided by evidence-based methods used in the demonstration of Bright IDEAS	Amount and type of pre-training reading, e.g., peer-reviewed journal articles
3. Bright IDEAS is a new intervention for most practitioners New psychosocial skills need to be integrated into the clinical workflow	A.Provide training and case mentorship to help providers learn the intervention within a team-based care approach	Training videos and practice working through an in-person challenge
4. Implementation of new clinical interventions is a combination of individual provider and institutional adoption Lack of institutional support and post-training participation reduces the likelihood of sustained individual adoption	Create training agreements regarding institutional support	Letter of supervisor support required for attendance (participant expectations outlined in letter). In pediatric oncology, psychosocial practitioners can independently adopt evidence-based interventions. Institutional support meant that there was visible buy-in to support their adoption of this new intervention. The letter signaled an intention-to-adopt expectation associated with the training vs. a continuing-education mindset so they could get a free trip to a conference.

**Table 2:** Bright IDEAS training adaptation for pediatric oncology practice.

**Initial training concerns**	**Adaptive training modifications**	**Rationale for modification**
**Need #1**. Identify caregivers in distress who might benefit most from Bright IDEAS		
Training workshop role-play was based on personal challenge making an attendee's translation of the psychosocial intervention steps into clinical care less intuitive	**Changed case study role play:** Training participants practiced the intervention using self-identified patient and/or family-focused case studies typical of their every-day practice	Make the clinical relevance of the Bright IDEAS intervention more evident
**Need #2**. Promote knowledge translation of evidence-base for Bright IDEAS		
Pre-workshop study materials relied on scientific evidence/papers that were perceived as too research intensive; participants believed the intervention could only be provided with scientific rigor	**Changed learning modality:** Participants watched online training videos and practiced working through a familiar clinical case challenge	Make the background information delivery more compatible with learning preferences for clinical practitioners thereby making it easier to acquire basic knowledge about the intervention
Workshop training was too research-focused and burdensome (i.e., like a study protocol) in its presentation of how to implement Bright IDEAS in clinical practice	**Added minimum intervention guidance:** Created a “Bright IDEAS essential elements” handout for practitioners to simplify the process	Distill the core elements of the intervention into a simple format so the clinical applicability of the Bright IDEAS intervention for real-world practice is more transparent
**Need #3**. Facilitate the integration of Bright IDEAS into clinical workflow		
Incorporating the Bright IDEAS intervention into the clinical workflow was not clearly evident	**Added clinical workflow guidance:** Included clinical workflow role plays and tips based on the experiences of practicing clinicians	Make the clinical compatibility of the Bright IDEAS intervention with real-world practice more transparent
**Need #4**. Ensure institutional support of Bright IDEAS to promote implementation and sustain adoption		
Clinicians registered for Bright IDEAS training program as individuals, without necessarily having institutional support for implementing the program at their home institution. Participation in the required post-workshop training component was sub-optimal	**Required institutional support:** A letter of supervisor support (with participant expectations outlined) was required for program registration and attendance. In pediatric oncology, psychosocial practitioners can independently adopt evidence-based interventions. Institutional support meant that there was visible buy-in to support their adoption of this new intervention. The letter signaled an intention-to-adopt expectation associated with the training vs. a continuing-education mindset so they could get a free trip to a conference.	Emphasize managerial support of training and follow-up consultation calls to foster an environment conducive to clinical adoption of Bright IDEAS

Bright IDEAS training participants were primarily female (91%) and from academic medical centers (82%). The majority of practitioners were social workers (47%) or psychologists (39%) (Table 3). Measures of Bright IDEAS use and satisfaction through 12 months following training suggest that professionals who received the training would recommend Bright IDEAS to a colleague remained strong through 12 months and improved after adaptations to the Bright IDEAS training program [9.11 vs. 8.38 (*p* < 0.001) on a 1 to 10 scale, where 10 = “extremely likely”]. Intervention usage, as measured by the mean number of clients to whom Bright IDEAS was delivered, also improved following adaptations to the Bright IDEAS training program at both 6 months (5.67 vs. 4.01, *p* < 0.001) and at 12 months (9.04 vs. 6.31, *p* = ns; Table 4).

**Table 3:** Characteristics of Bright IDEAS training participants.

**Measure**	**No. (%) of trainees who participated in the Bright IDEAS training program**			
	**Pre-adaptation (*N* = 159)**	**Post-adaptation (*N* = 50)**	**Overall (*N* = 209)**	***P*** **-value**
**Gender**				
Female	147 (92.5%)	50 (100.0%)	197 (94.3%)	0.045
Male	12 (7.5%)	0 (0.0%)	12 (5.7%)	
**Race**				
Caucasian	136 (86.1%)	36 (73.5%)	172 (83.1%)	0.28
African American	7 (4.4%)	4 (8.2%)	11 (5.3%)	
Asian/Pacific Islander	7 (4.4%)	4 (8.2%)	11 (5.3%)	
Mixed	3 (1.9%)	3 (6.1%)	6 (2.9%)	
Other	5 (3.2%)	2 (4.1%)	7 (3.4%)	
**Primary professional discipline**				
Social worker	79 (50.0%)	22 (44.0%)	101 (48.6%)	0.043
Psychologist	61 (38.6%)	20 (40.0%)	81 (38.9%)	
Nurse	11 (7.0%)	3 (6.0%)	14 (6.7%)	
Physician	0 (0.0%)	3 (6.0%)	3 (1.4%)	
Other health profession	7 (4.4%)	2 (4.0%)	9 (4.3%)	
**Years of pediatric oncology experience**				
0–2 years	44 (28.2%)	21 (42.0%)	65 (31.6%)	0.26
3–5 years	37 (23.7%)	9 (18.0%)	46 (22.3%)	
6–10 years	30 (19.2%)	6 (12.0%)	36 (17.5%)	
Over 10 years	45 (28.8%)	14 (28.0%)	59 (28.6%)	

Source: Data collected at time of Bright IDEAS training registration. N missing was 1.4% (N = 3) or fewer, depending upon the question.

**Table 4:** Adoption of Bright IDEAS in pediatric oncology practice among training participants.

	**Measures of use and satisfaction, mean (s.d.)**							
	**6-months post training** ^*^				**12-months post training** ^**^			
**Measure**	**Pre-adaptation**	**Post-adaptation**	**Overall**	***P*** **-value**	**Pre-adaptation**	**Post-adaptation**	**Overall**	***P*** **-value**
	**(*N* = 159)**	**(*N* = 50)**	**(*N* = 209)**		**(*N* = 159)**	**(*N* = 50)**	**(*N* = 209)**	
No. of clients to whom Bright IDEAS has been delivered (mean)	4.01 (2.35)	5.67 (2.37)	4.36 (2.44)	<0.001	6.31 (4.38)	9.04 (7.43)	6.81 (5.14)	0.08
“Likelihood I will recommend Bright IDEAS to a colleague.” (scale = 1 to 10, where 10 = “extremely likely”)	8.37 (1.66)	8.60 (1.66)	8.42 (1.66)	0.45	8.23 (1.83)	9.11 (1.09)	8.38 (1.75)	0.001

Source: Online surveys administered 6 and 12 months after the initial in-person training session.

* N missing ranged between 14.4% (N = 30) to 18.6% (N = 39), depending upon the question.

** N missing ranged between 27.8% (N = 58) and 28.7% (N = 60), depending upon the question.

The most common situations endorsed as barriers were: “I lack time”, “Incorporating Bright IDEAS into my clinical workflow”, and “Client compliance issues”. “Lack of consensus of professional guidelines”, “Reimbursement and/or insurance issues”, and “Lack of experience” were reported as barriers in less than 10% of trainees (Table 5). Overall, the rank order of surveyed barriers and their perceived magnitude did not change appreciably after adaptation. The exceptions were: “I lack time” which decreased post-adaptation at the 12-month assessment (from 63 to 41% reporting as a barrier, *p* < 0.05); and “Lack of experience” which increased (from 3 to 15%, *p* < 0.05).

**Table 5:** Reported barriers to Bright IDEAS adoption, implementation and maintenance in pediatric oncology practice.

**Measures of implementation barriers**	**No. (%) Reporting “Yes, this measure is a barrier.”**							
	**6-months post training** ^*^				**12-months post training** ^**^			
	**Pre-adaptation**	**Post-adaptation**	**Overall**	***P*** **-value**	**Pre-adaptation**	**Post-adaptation**	**Overall**	***P*** **-value**
	**(*N* = 159)**	**(*N* = 50)**	**(*N* = 209)**		**(*N* = 159)**	**(*N* = 50)**	**(*N* = 209)**	
I lack time (to assess/counsel clients)	83 (59.7%)	23 (57.5%)	106 (59.2%)	0.80	77 (63.1%)	11 (40.7%)	88 (59.1%)	0.03
Incorporating Bright IDEAS into routine care (clinical work flow)	68 (48.9%)	17 (42.5%)	85 (47.5%)	0.47	72 (59.0%)	16 (59.3%)	88 (59.1%)	0.98
Client compliance issues	53 (38.1%)	15 (37.5%)	68 (38.0%)	0.94	56 (45.9%)	13 (48.1%)	69 (46.3%)	0.83
Bright Ideas takes too much time	31 (22.3%)	6 (15.0%)	37 (20.7%)	0.31	28 (23.0%)	7 (25.9%)	35 (23.5%)	0.74
Lack of opportunity (clients)	30 (21.6%)	10 (25.0%)	40 (22.3%)	0.65	30 (24.6%)	6 (22.2%)	36 (24.2%)	0.79
Lack of administrative support	11 (7.9%)	3 (7.5%)	14 (7.8%)	0.93	13 (10.7%)	2 (7.4%)	15 (10.1%)	0.61
Lack of experience	10 (7.2%)	3 (7.5%)	13 (7.3%)	0.95	4 (3.3%)	4 (14.8%)	8 (5.4%)	0.02
Reimbursement and/or insurance issues	10 (7.2%)	1 (2.5%)	11 (6.1%)	0.28	7 (5.7%)	1 (3.7%)	8 (5.4%)	0.67
Lack of consensus of professional guidelines	2 (1.4%)	0 (0.0%)	2 (1.1%)	0.45	1 (0.8%)	0 (0.0%)	1 (0.7%)	0.64

Source: Online surveys administered 6 and 12 months after the initial in-person training session.

* N missing was 14.4% (N = 30).

** N missing was 28.7% (N = 60).

### Qualitative findings

Table 6 summarizes perceptions from the in-depth interviews about implementing Bright IDEAS into pediatric oncology practice. Data appeared to become redundant following the 23rd interview during the pre-adaptive phase and following the 11th interview during the post-adaptive phase. All authors agreed that no unique responses were emerging within the data and that saturation had been reached (18, 24). As practitioners had already agreed to participate, seven more interviews were completed during the pre-adaptation phase and three more during the post-adaptation phase. As no interview data was omitted, reported results reflects all the interview data. Representative quotes are provided to support the rationale for program adaptation of identified forms of Bright IDEAS to better align the program with clinical practice. The following further compares intervention perceptions before, and after, adaptations to the Bright IDEAS training program using three key constructs of the RE-AIM framework: adoption, implementation, and maintenance.

**Table 6:** Perceptions about implementing the Bright IDEAS program in pediatric oncology practice among training participants before and after program adaptation.

**RE-AIM dimension**	**Bright IDEAS forms -focus of adaptation**	**Representative quotes**	
		**Initial curriculum (*N* = 33) (modeled from clinical research protocol)**	**Adapted curriculum (*n* = 14) (tailored for real-world clinical setting)**
**Adoption**. Bright IDEAS is adopted by clinicians and practice settings	Identification of appropriate patient profile	“Individuals or parents that are ready to engage in problem-solving vs. they still need some initial time to process the diagnosis and get through potentially the aspect of degrees of denial at first, I would give them that time before I would embark on utilizing the Bright IDEAS paradigm.”	“The most successful family that I have used this [Bright IDEAS] with were parents who were very psychologically minded, had pursued therapy themselves throughout the years, and really were asking for psychology involvement at the time of their child's diagnosis.”
		“When I'm talking with families, if there's some anxiety or stress, or the parent is critical about something, I sort of put them on my sort of mental list of okay, this might be a good idea for Bright IDEAS.” “I don't have, and my colleague doesn't have the ability to really sit down with families and say, you know, “This is important, and, and we want you to use these tools.”	I pick parents that I feel are highly anxious…they're searching for some type of sense of control. I feel like using the form and guiding them through it [Bright IDEAS] gives them that.
**Implementation**. Bright IDEAS is implemented consistently into clinical workflow	Implementing Bright IDEAS in the clinical setting	“If I didn't have the forms with me, and I was meeting a family spur of the moment, I didn't have time to…run back to my office and get the forms…”	“It's [Bright IDEAS] very flexible in the way that we don't have to abide by a certain number of sessions …we can just use it however we see fit for every single family. So, I think that it is seamlessly worked into the work that I do...”
		“I may do a consult and then they're discharged and – and they don't necessarily come back to clinic.”	“I have been able to implement Bright IDEAS in all different settings. So, I have done it inpatient, I have done it on the outpatient side and certainly done it in clinic as well. It's possible”.
**Maintenance**. Bright IDEAS is maintained over time	Need for institutional support	“I think that something like Bright IDEAS is likely to be more popular and more widely disseminated at an institution where there is a big psychosocial team and a lot of buy-in...” “It's definitely not something that I get that's getting publicity and, you know, I don't know if it would change if it was more widely known by, like, attendings and the broader medical team, but if that would somehow change how well affected it is or how well known it is”	“So I think, having maybe more institutional support or I don't know if there was, you know any sort of incentive for providers to use it [Bright IDEAS].” “I'll tell you that I don't think I would have been able to go [Bright IDEAS training] had I not been reimbursed or had most of it not been reimbursed. If I had the financial support for ongoing training, that would be helpful.”

Source: Qualitative interviews (N = 47): pre-adaptation (n = 33) and post-adaptation (n = 14).

#### Bright IDEAS adoption

Overall, there was general agreement that Bright IDEAS (referred to as “BI” in the quotes) was initially adopted, or not, at the independent discretion of the practitioner, with no institutional oversight. This resulted in practitioners creating their own methods for identifying clients they thought appropriate for the intervention. For example, a social worker described the type of client for the intervention this way: “I pretty much just think about how they're dealing with particular problems they seem to be having and deciding on my own whether or not I think BI would be a good intervention for them.” Additionally, as one psychologist noted, “some of it is based on how the family presents, and how they buy in [BI].”

After adaptations to the Bright IDEAS training program, the revised intervention materials were noted as useful with their clients and helped to organize the practitioner's clinical work. For example, a social worker stated, “I usually keep the worksheets and I have them take a picture with their phone of the action plan.” The profile of the ideal patient was identified by one psychologist as:

“Patients that have a lot of stressors that tends to be ongoing. So, that could either be a diagnosis and they've just started treatment, or that could be longstanding, strained relationships with their family or their partner. In addition to that, I would say families that don't have a lot of social support specifically, family or social support are good candidates for BI.”

#### Bright IDEAS implementation

In general, practitioners found difficulty with providing numerous written intervention materials with clients and sometimes “forgot” about using BI. For example, clients seemed overwhelmed with the new cancer diagnosis and unable to process a new resource, demonstrated by one psychologist's experience from a client, “Oh my gosh. Are you kidding me? They just gave me a calendar for medication and now you want me to write some things down?”

Additionally, it was observed that use of BI was inconsistent across clients. For example, a social worker stated, “I haven't been able to use this (BI) as frequently as I would have hoped.” Comparatively, a psychologist stated, “I have been offering it (BI). I go, introduce myself to families at the time of diagnosis and introduce the program as a support tool, and then follow-up after 4 weeks, you know the next time they're admitted, and then kind of offer the program at that point and time. It's standard just offering it to everybody regardless.” However, some practitioners found it difficult to fully deliver the prescribed five steps of BI as noted by one psychologist, “I think when we were trying to track things at the very beginning and be able to report back every month what was happening it felt just so much more rigid and made it difficult for the family to keep up with all of it.”

After adaptations to the Bright IDEAS training program, Bright IDEAS tended to be used in a greater variety of clinical situations. Specifically, a psychologist reported, “I have been able to implement BI in all different settings. So, I have done it inpatient, I have done it on the outpatient side and, and certainly done it in clinic as well. It is possible.” Similarly, a social worker recounted, “I work for a nonprofit and we actually are a community-based organization. So, we go into the patient's house…and meet with them in their environment to discuss their problems…using BI.”

#### Bright IDEAS maintenance

Plans to maintain the use of Bright IDEAS over time varied between practitioners; variability was partially explained by the local health system context. One psychologist noted,

“Our division of labor, will all be changing because we are one division that serves two hospitals and so once we all are under one program, the way that we provide psychosocial services will be changing, and our goal is that BI in the long term becomes part of a process where we offer it [BI] to everybody but that families who are identified as higher risk factors for all sorts of issues associated with the diagnosis, managing the diagnosis, et cetera, will be offered that program with a little bit more of a push.”

There was also a psychologist that took the initiative to train other practitioners, demonstrated by the exemplar,

“I have actually trained all of my students here. I trained my counterpart at our center as well as the social worker over there, and then we had other staff members here at our children's hospital, like we had a child life specialist ask to sit in on training, social work asked to sit in on training, so they have all been trained here already, and then additionally, we'll be kind of continually training students as they come through with us and I have also trained our child and adolescent psychiatry team who function in an outpatient mental health clinic, so they – we trained them as well.”

Comparatively, some practitioners mentioned being the “only one” at their institution trained in the intervention and recognized the difficulty in maintaining use of Bright IDEAS in their absence.

After adaptations to the Bright IDEAS training program, practitioners discussed ways in which they plan to continue to use Bright IDEAS over time. For example, one social worker shared, “I just consider it [BI] to be another very useful tool in my toolbox to use. I plan to just keep using it for families that clearly will benefit from it.” The sentiment was also expressed as, “I feel like it [BI] is something I'm going to always continue to use. If I notice that there are certain participants or patients, I have that would really benefit from having the goals.” Additionally, there were examples of planned internal training, “So, on my team there's two other people, a social worker, and a counselor. The plan is to teach them BI.”

## Discussion

To the authors' knowledge, this is the first systematic multi-methods evaluation of the functions and forms of an NCI EBCCP-recognized intervention and its dissemination into clinical oncology practice. This study identifies the core functions and forms of a psychosocial intervention to address barriers to adoption, implementation, and maintenance of Bright IDEAS in real-world settings. Based on feedback from trainees, adaptations to the Bright IDEAS training program were made to foster a more pragmatic approach to intervention delivery and sustainment. The new training model fostered expanded use and acceptance of Bright IDEAS at the individual level.

The goal of the grant was to increase national awareness of Bright IDEAS, train providers on how to deliver Bright IDEAS, and facilitate adoption amongst the approximately 200 pediatric oncology centers operating in North America, as well as elucidate barriers to adoption, implementation, and maintenance in diverse real-world pediatric oncology practice settings. There was a clear distinction between the two training groups, indicating a positive response to the adaptations. We believe the difference between the two groups could be partially explained by the fact that behavioral interventions are more difficult to define and standardize because of the inherent interactivity with local client characteristics, preferences, and behaviors (25). Although Bright IDEAS has been proven to be efficacious for more than 20 years, factors affecting real-world application had not been studied.

Historically, the translation of tested interventions into clinical practice is limited by the inherent tension between intervention development and efficacy testing in the context of tightly-controlled explanatory trials and implementation in real-world settings (26). However, successful implementation of evidence-based interventions requires flexibility in treatment delivery based on clinical context. Scaling and sustainability of evidence-based programs often requires a trade-off between fidelity to the trial protocol and feasibility in a real-world clinical setting (27). To address this gap, stakeholder-informed adaptations to the training program as the intervention was being nationally scaled promoted flexibility in intervention delivery and feasibility based on dynamics of diverse clinical settings.

Implementation science has historically endorsed intervention permanence – i.e., once the evidence-base has been established for an intervention then practitioners can directly proceed to implementation, scale-up and sustainability. However, Chambers and Norton (28) posit that concerns around program drift and requirements for intervention permanence have not served implementation sciences well because it may hinder translation into real-world clinical practice (28). This study is an exemplar of an iterative approach to advance implementation science which responds to the call by revisiting the training protocol and delivery of Bright IDEAS in the clinical setting and acknowledging that interventions are not static events, rather they are dynamic in nature requiring continuing adaptations to meet the numerous demands of clinicians and the ever-changing context of the setting in which they are deployed.

This innovative approach to identifying standard core functions and how the forms were adapted to match practice characteristics was key in understanding practitioner needs and environmental factors. For example, we advised that Bright IDEAS not be formally introduced to families with newly diagnosed children until at least 4 weeks later, precisely because of a “not now” response upon initial implementation (1, 2, 4, 6). Lau et al. (29) observed similar results when examining adolescents and young adults' perspectives on facilitators and barriers to utilization of psychosocial programs and found that “starting something new” could be a significant barrier to utilization.

The current study revealed moderating factors that may affect adoption more broadly. This finding is not surprising as Greenhalgh et al. (30) noted that standard attributes of the intervention will not ensure adoption alone (30). Rather, the interaction among the intervention, intended adopters, and a particular context is what determines adoption rate (30). In this study, adaptations to the Bright IDEAS training program considered both practitioner experience and local setting. While there was improved adoption with adaptation, there is still opportunity for progress. Chambers and Norton (28) describe the necessary fit between interventions and their settings and suggest ongoing learning about optimal intervention delivery over time (28). Gathering feedback across diverse clinical settings should be planned as an iterative process that accounts for evolving methods of care and practice settings (31, 32).

The field has an enormous opportunity within the context of dissemination and implementation research to elucidate a full science of intervention adaptation (28). This study adds to empirical evidence by systematically collecting information on the impact of adaptation to practitioners and used this information to extend the knowledge base of implementation of evidence-based practices as well as ongoing improvement of the Bright IDEAS.

The path toward sustained maintenance of Bright IDEAS in clinical settings remains to be identified. While there were practitioners that intentionally trained colleagues or fellows, such training efforts were rarely supported by institutional leadership beyond attending training. Generalizable lessons learned underscore the importance of continual stakeholder engagement and administrative assistance to ensure long-term maintenance.

## Limitations

While the aim of this study was to provide lessons learned to inform dissemination and sustainability planning for other psychosocial interventions, there are limitations that should be noted. First, results may be difficult to generalize to other diseases as practitioners were recruited from national organizations with a focus on pediatric and adolescent cancer. Another limitation is the possibility of social desirability bias. That is, some providers may have responded to questions in a manner they thought consistent with the research aims of the project. Future research with other stakeholders, such as institutional leadership and members of patient treatment teams, would be valuable to understanding factors that affect the dissemination and implementation process in the clinical setting.

The adaptations made to the psychosocial intervention training workshop presented in this study can help to bridge the science-to-service gap in mental health care and may provide important information regarding facilitators and barriers to implementation for other mental health researchers and implementation scientists. Moreover, may also be effective for the implementation of other psychosocial interventions or innovations in psychiatric care for patients with cancer, survivors of cancer, and for caregivers of those with cancer.

## Summary

This multi-methods evaluation of a national training program highlights some of the issues psychosocial providers face when translating a new evidence-based intervention from research to practice settings, and the steps that can be taken to improve implementation. Further, attention to the fit between characteristics of an intervention and the clinical setting and the availability of resources, and knowledge of potential implementers is critical for informing an implementation process that capitalizes on facilitators and “works around” barriers. For busy psychologists and social workers, we found that a blend of strategies that helps to increase compatibility with existing organizational structures is critical for implementation.

Future pediatric oncology-based psychosocial interventions should build on the current focus of addressing adoption, implementation, and maintenance issues at the design stage of interventions when trials are first initiated (33). In addition, teams should explore adaptive dissemination strategies that aim to evolve to meet the dynamic nature of the clinical environment. Maintenance requires integration of research-tested protocols tailored for easy incorporation into routine clinical workflow. Longitudinal follow-up post training is imperative to ensuring the maintenance of an intervention; otherwise, “out of sight, out of mind” is inevitable.

## Data availability statement

The raw data supporting the conclusions of this article will be made available by the authors, without undue reservation.

## Ethics statement

The studies involving human participants were reviewed and approved by the Colorado Combined Institutional Review Board (COMIRB). Written informed consent for participation was not required for this study in accordance with the national legislation and the institutional requirements.

## Author contributions

OS, RN, and EM conceived and designed the experiment. DM, MV, and DF performed the experiments. DM, SB, and DF analyzed the data. OS, RN, MV, and DF contributed materials/analysis tools. DM and EM wrote the paper. OS and RN obtained the funding. All authors contributed to the article and approved the submitted version.

## Funding

This study was funded by the National Institutes of Health and the National Cancer Institute for funding NIH Grant R01 CA 159013 and R25 CA65520.

## Conflict of interest

The authors declare that the research was conducted in the absence of any commercial or financial relationships that could be construed as a potential conflict of interest.

## Publisher's note

All claims expressed in this article are solely those of the authors and do not necessarily represent those of their affiliated organizations, or those of the publisher, the editors and the reviewers. Any product that may be evaluated in this article, or claim that may be made by its manufacturer, is not guaranteed or endorsed by the publisher.

## Abbreviations

APHON: Association of Pediatric Hematology/Oncology Nurses
APOSW: Association of Pediatric Oncology Social Workers
COG: Children's Oncology Group
COMIRB: Colorado Combined Institutional Review Board (COMIRB)
EBCCP: Evidence-Based Cancer Control Programs
NCI: National Cancer Institute
SPP: Society of Pediatric Psychology.
