# Supplementary material for: Core functions and forms of Bright IDEAS: A multi-methods evaluation of the adoption of an evidence-based psychosocial training program through iterative adaptation
Source: Front Health Serv. 2022 Nov 4;2:928580. doi: 10.3389/frhs.2022.928580 (PMC10012676; doi:10.3389/frhs.2022.928580)
Supplement: Supplementary file 1 [file Table_1.DOCX]

Supplementary Material

# Supplementary Data (Interview guide)

Bright IDEAS Trained Providers Interview Guide

Interviewer Initials: __________

Study Participant Number: __________

Location: Phone

Date: ___________

Welcome:

Thank you for agreeing to participate in this interview where we will explore the ways Bright IDEAS has been implemented and could possibly be sustained as an evidence-based intervention in a clinical setting at your institution and more broadly.

Introductions

My name is Demetria McNeal and I am with the University of Colorado. We are working with Dr. Sahler of the University of Rochester and Dr. Noll of the University of Pittsburgh on this research project to evaluate and learn from your experiences. We are trying to understand how Bright IDEAS, a problem-solving skills training (PSST) intervention for caregivers of children diagnosed with cancer, has been adopted, adapted, implemented and can potentially be sustained in a clinical setting.

In this interview, when I say the word adoption I mean individuals or institutions deciding to try out Bright IDEAS with his/her clients.

In this interview, when I say the word implementation I mean how Bright IDEAS is actually delivered in a clinical setting.

In this interview, when I say the word sustainability I mean the extent to which institutions may continue to deliver Bright IDEAS long-term.

I want you to know that there are no right or wrong answers to any of the questions I will ask you today. Every practitioner’s experiences and opinions are important and we want to hear a range of opinions on the questions we’ll be asking. The way our research teams looks at it, you are the expert. We value your opinions and want you to know that what professionals tell us during these discussions may help families with a child diagnosed with cancer get the support they need. When you have completed the interview, I will collect some information from you so that I can mail you a gift card to thank you for participating in the interview. Do you have any questions?

This interview should take around 1 hour. All responses will be kept private and confidential. To be able to analyze the information we collect from practitioners, it is helpful to record the interview. But the transcript will not have people’s names included. Is it okay with you for me to record our discussion today? [IF REFUSES RECORDING, INTERVIEWER WILL TAKE DETAILED NOTES INSTEAD]

Please feel comfortable to ask questions at any time during the interview. Can I confirm that you are okay talking with me about these topics today?

Interviewer: By checking this box ☐ the person consents to this interview.

START RECORDER AND STATE: “Bright IDEAS dissemination grant, [today’s date], and study participant number [#]”

Interview Questions

1. Let’s start with some basics

a. What is your role?

b. In what type of institution do you work?

Probe: Is it an academic center? Clinic? Cancer center?

2. How many client cases do you typically manage in a week? About how many of those cases have a child newly diagnosed with cancer?

a. Probe: How many children newly diagnosed with cancer are treated at your institution annually?

b. Probe: About how many clients have you used Bright IDEAS with since your training?

3. How did you first learn about Bright IDEAS?

a. Probe: How long have you known about Bright IDEAS?

b. Probe: Did you know colleagues using Bright IDEAS before you attended the training workshop?

Bright IDEAS was designed to be a resource for individuals to learn to use problem-solving skills to cope effectively with stress and emotional distress when managing a newly diagnosed child with cancer. Now thinking about other similar resources that might be available for you to offer to parents that have a child newly diagnosed with cancer, (4) which ones are you or your institution using?

a. Probe: Is [insert name of resource(s) stated by participant] part of an established protocol? Are you aware of whether [insert name of resource stated by participant] is evidence-based?

b. Probe: In what ways does your institution support you in using [insert name of resource stated by participant] with your clients?

5. What has been your experience with delivering the Bright IDEAS program to your clients?

a. Probe: Was there anything that you found helpful when delivering Bright IDEAS?

b. Probe: Was there anything that you found difficult about delivering Bright IDEAS?

Now, I’d like to discuss the adoption of Bright IDEAS. If you recall, adoption means individuals or institutions choosing or deciding to try out Bright IDEAS with his/her clients. Bright IDEAS has

been researched as a successful evidence-based intervention for over 20 years, yet it has not been broadly adopted into clinical practice. One reason cited is that few practitioners have heard of or are

aware of it being used by colleagues. We’d like your opinion on some of the reasons that may be the case.

6. Please tell me some of the reasons that you think Bright IDEAS is

not more well-known by mental health professionals in childhood cancer clinical settings?

7. Why did you apply for Bright IDEAS training?

8. Which factors influenced your decision to receive training in Bright IDEAS?

a. Probe (sociopolitical): How would someone would get a new evidence-based practice approved for use in your institution? Did you have to do that to be able to use Bright IDEAS?

b. Probe (context): How Bright IDEAS compare to the other resources that you also use, or considered using, with parents with a child diagnosed with cancer?

c. Probe (innovation): Would you consider Bright IDEAS to be a completely new approach to working with parents with a child newly diagnosed with cancer?

[Interviewer notes: If yes, ask: how so? If no, ask: what is it most similar to that you have used or are more familiar with?]

9. How has your institution supported your ability to deliver Bright IDEAS?

a. Probe: How has using Bright IDEAS with a parent impeded working with your colleagues?

Now, I’d like to talk about your experiences with implementing Bright IDEAS. By implementation, I mean how Bright IDEAS is actually delivered in a clinical practice.

10. Please tell me about the ways you deliver Bright IDEAS to your clients?

a. Probe: How do you select appropriate clients for Bright IDEAS?

b. Probe: How do you determine the number of sessions? How do you determine how long the sessions should be?

c. Probe: How do you know when you are ‘done’ or that Bright IDEAS ‘is working’? In other words, what is your idea of what success would look like? Have you had ‘success stories’ with Bright IDEAS?

[Interviewer notes: If yes, ask: In what ways was the successful parent or patient different from the parent or patient that was not as successful?]

11. What is it like when you discuss Bright IDEAS with a client?

a. Probe: Does the discussion tend to go as planned?

b. Probe: What tends to hinder the discussion?

c. Probe: What helps the discussion?

Now, I’d like to talk about your experiences with materials. Bright IDEAS have many resources available to clinicians once training is complete. Thinking about the Bright IDEAS materials

available to you and your clients, we are going to focus on how you have been able to utilize them in a clinical setting.

13. What has been your experience with utilizing the Bright IDEAS…

a. manual ?

b. worksheets ?

c. booklet?

d. website?

e. bookmark?

For each material, probe:

Did you use the material for your own resource? With clients or families? Or both?

What did you find helpful about incorporating the resources into your delivery of Bright IDEAS PSST?

What did you find difficult about incorporating the resources into your delivery of BRIGHT IDEAS PSST ?

17. Some professionals who have been trained in the Bright IDEAS program have recommended we help connect trainees with other trained colleagues via a social media platform. This might be like a Bright IDEAS user group and FAQ blog posting.

a. What do you think of this idea?

b. [For those trained after it is available] Did you use it? What did you find helpful or that you liked? Was there anything that you found difficult or that you disliked?

Now, I’d like to talk about the sustainability of Bright IDEAS. By sustainability, I mean the extent to which institutions may continue to deliver Bright IDEAS long-term.

18. Please tell me about any future plans you have to deliver Bright IDEAS to your clients?

19. What factors might influence your (dis) continuation of Bright IDEAS?

a. Probe: How significant a role does [insert factors stated by participant] play in determining your future use of Bright IDEAS?

20. In an ideal clinical setting, what would have to happen to make Bright IDEAS a useful tool for you to use?

a. Probe: What type of client?

b. Probe: What type of (institutional) support would you need?

c. Probe: Anything else you would need?

21. If you were going to redesign Bright IDEAS so that you could use it with your clients every time that you thought it would be appropriate, how would you change it?

22. What are your intentions for using Bright IDEAS in the future (in the next 6 months)?

[Interviewer notes: If limited use stated, ask: What would you need to be able to use it fully in the future?]

23. Do you think your institution intends to encourage the use of Bright IDEAS in the near future (in the next 6 months)?

a. Probe: Among your peers?

b. Probe: Maybe implement organizational changes?

24. In what ways could your institution increase the use of Bright IDEAS?

25. What advice would you give to a practitioner that is thinking about implementing Bright IDEAS in their institution?

Thank you for that information. It is very helpful. We are nearly done with this interview. Now I would like to get some suggestions on how to improve the Bright IDEAS training program.

26. What parts of the Bright IDEAS training would you change?

a. Probe: In what ways would those changes improve the training?

[Interviewer notes: probe for content, reach, and leadership support]

Thank you for all that feedback! Those are all my questions for today. But before we end, I do want to ask you:

27. What have we missed? What else should we discuss?

Thank you so much for your time and for sharing your ideas with us! Now I will collect information to provide you with your $25 VISA gift card for your participation today.

[Collect information for $25 gift card]

Email address: _______________________________________or,

Mailing address: _______________________________________

_______________________________________

_______________________________________

_______________________________________
